# Health Tract, No. 233

**Published:** 1865

**Authors:** 


### RESTLESSNESS,

Whether of body or mind, is a great calamity; it is a disease as to both. Forty years ago I saw the son of "the great commoner" walking in the inclosure of a lunatic asylum, and learned from an old school-mate, the other day, that "Theodore was walking still." Quiet, inoffensive, the forty years' journey is not yet finished.

I know a man with his millions; he earned them all by his own exertions; he sat him down to rest; but the mind, by its intense application, had acquired such a momentum, that the effort to stop it, made a wreck of it. It is always a dangerous experiment to retire abruptly from active business. That mind had to work on something; it could not stand "beating the air," and dealing with "vain thoughts;" it was necessary that it should have a tangible object; and for ten years that poor man has been sitting at his table, counting his gold and arranging it in piles, first on one side of the table and then on the other. He loves the occupation; he knows no other happiness, and has no higher joy; there he is, from one year's end to another, in a Massachusetts asylum, behind its grated windows, away from friend and home, and wife and children, counting his dollars; he gets no better, gets no worse. He eats and drinks and sleeps, then sits down to count and pile up, with as much apparent zest as if it were a new thing to a little child. Not only is his wealth lost to him, but life itself, and the great future. If we could know that it would be a mere blank, there would be some relief to the inquiring mind; but when disability comes, unfitting a man for any of life's duties, and for the great preparation needed to secure

"A mansion in the skies,"

is brought on by a prostitution of the noblest powers of the immortal mind by making gold

"A vile idolatry,"

there can not be an exemption from that "fearful looking for of judgment," which is the heritage of those who neglect the great salvation. But while we take satisfaction in reference to this, as well as other important questions of the same class, in the reflection, so sweet, so comforting, so worth all worlds, "Shall not the judge of all the earth do right?" let each reader for himself learn to follow the pursuits of life with a moderate, a wise ambition; "using the things of this world as not abusing them;" regarding them as a means and not an end—the means of having an opportunity of securing a treasure in heaven which moth and rust can not corrupt, nor thieves break through and steal.

